# Human Muscle Progenitor Cells Overexpressing Neurotrophic Factors Improve Neuronal Regeneration in a Sciatic Nerve Injury Mouse Model

**DOI:** 10.3389/fnins.2019.00151

**Published:** 2019-02-27

**Authors:** Reut Guy, Frida Grynspan, Tali Ben-Zur, Avraham Panski, Ron Lamdan, Uri Danon, David Yaffe, Daniel Offen

**Affiliations:** ^1^Laboratory of Neuroscience, Felsenstein Medical Research Center, Sackler Faculty of Medicine, Tel Aviv University, Tel Aviv, Israel; ^2^Stem Cell Medicine Ltd., Jerusalem, Israel; ^3^Department of Orthopedic Surgery, Hadassah-Hebrew University Medical Center, Jerusalem, Israel; ^4^Department of Molecular Cell Biology, Weizmann Institute of Science, Rehovot, Israel

**Keywords:** human muscle progenitor cells, neurotrophic factors, BDNF, GDNF, VEGF, IGF-1, peripheral nerve injury, sciatic nerve injury

## Abstract

The peripheral nervous system has an intrinsic ability to regenerate after injury. However, this process is slow, incomplete, and often accompanied by disturbing motor and sensory consequences. Sciatic nerve injury (SNI), which is the most common model for studying peripheral nerve injury, is characterized by damage to both motor and sensory fibers. The main goal of this study is to examine the feasibility of administration of human muscle progenitor cells (hMPCs) overexpressing neurotrophic factor (NTF) genes, known to protect peripheral neurons and enhance axon regeneration and functional recovery, to ameliorate motoric and sensory deficits in SNI mouse model. To this end, hMPCs were isolated from a human muscle biopsy, and manipulated to ectopically express brain-derived neurotrophic factor (BDNF), glial-cell-line-derived neurotrophic factor (GDNF), vascular endothelial growth factor (VEGF), and insulin-like growth factor (IGF-1). These hMPC-NTF were transplanted into the gastrocnemius muscle of mice after SNI, and motor and sensory functions of the mice were assessed using the CatWalk XT system and the hot plate test. ELISA analysis showed that genetically manipulated hMPC-NTF express significant amounts of BDNF, GDNF, VEGF, or IGF-1. Transplantation of 3 × 10^6^ hMPC-NTF was shown to improve motor function and gait pattern in mice following SNI surgery, as indicated by the CatWalk XT system 7 days post-surgery. Moreover, using the hot-plate test, performed 6 days after surgery, the treated mice showed less sensory deficits, indicating a palliative effect of the treatment. ELISA analysis following transplantation demonstrated increased NTF expression levels in the gastrocnemius muscle of the treated mice, reinforcing the hypothesis that the observed positive effect was due to the transplantation of the genetically manipulated hMPC-NTF. These results show that genetically modified hMPC can alleviate both motoric and sensory deficits of SNI. The use of hMPC-NTF demonstrates the feasibility of a treatment paradigm, which may lead to rapid, high-quality healing of damaged peripheral nerves due to administration of hMPC. Our approach suggests a possible clinical application for the treatment of peripheral nerve injury.

## Introduction

Peripheral nerve injury can occur in daily life due to mechanical damage resulting from traffic accidents, sports, or surgery. Such injury poses challenges for patients ranging from minor discomfort to harming quality of life (Clin, 2015). Peripheral neurons have the ability to reactivate their intrinsic growth capacity and allow regeneration to occur following injury (Xu et al., 2013). Nevertheless, the clinical outcome of this regeneration is often incomplete, as expressed in symptoms such as poor and abnormal sensibility, deficient motor function, cold intolerance, and pain (Lundborg, 2000).

Sciatic nerve crush is one of the most common models for peripheral nerve injury. The sciatic nerve is the longest nerve in the human body, extending from the lower part of the spinal cord to the buttocks and down the legs (Glat et al., 2016). It comprises both motor and sensory fibers, therefore the sciatic nerve injury (SNI) model closely simulates general peripheral nerve damage, in a simple, reproducible manner.

Neurotrophic factors (NTFs), including brain-derived neurotrophic factor (BDNF), glial-cell-line-derived neurotrophic factor (GDNF), vascular endothelial growth factor (VEGF), and insulin-like growth factor 1 (IGF-1), are molecules which enhance growth and survivability of neurons. BDNF was found to be essential for peripheral nerve regeneration and remyelination after injury (Zhang et al., 2000). GDNF was shown to have a maintenance role for adult motor neurons (Naveilhan et al., 1997) and to prevent motor neuron degeneration following peripheral axotomy (Oppenheim et al., 1995; Yan et al., 1995; Hoozemans et al., 2009). VEGF was shown to support and enhance the growth of regenerating nerve fibers (Sondell et al., 2000; Lopes et al., 2011). IGF-1 was shown to exert important growth supporting effects on regenerating peripheral nerves (Hansson et al., 1986; Kanje et al., 1989; Sjöberg and Kanje, 1989). Unfortunately, these findings have not yet led to a clinical treatment improving peripheral nerve repair.

Muscle progenitor cells (MPC) are an easily accessible cell type, with well-characterized markers associated with various stages of differentiation (Sarig et al., 2010). MPC are also relatively simple to clone and manipulate in culture (Yaffe, 1968, 1969; Sarig et al., 2006). In a previous study, we showed that transplantation of a mixture of rat myogenic cell line L8, genetically modified to express and secrete BDNF, GDNF, IGF-1 or VEGF (each population expressing a single NTF) have a strong synergistic effect on the regeneration of a damaged sciatic nerve, in a rat model (Dadon-Nachum et al., 2012). The MPC mixture harboring together the four NTFs, was shown to accelerate recovery of motor function, preserved the compound muscle action potential, and inhibited degeneration of the neuromuscular junctions. We further showed that direct intramuscular administration of a mixture of lentiviral vectors expressing the four NTFs significantly improved the recovery of axonal function in a mouse model of SNI (Glat et al., 2016).

In the present study, we examined the effect of intramuscular administration of human muscle progenitor cells (hMPC) overexpressing the NTF genes - BDNF, GDNF, VEGF, and IGF-1, in a mouse model of SNI. We demonstrate that transplantation of the hMPC-NTF, 1 day after sciatic nerve crush, can speed and ameliorate natural neuronal regeneration.

## Materials and Methods

### Isolation of Primary Myoblasts

A human muscle biopsy (one patient; 2 cm^2^× 2 cm^2^) was collected by an orthopedic surgeon during surgery performed for reasons unrelated to the biopsy for the current research. A written informed consent was obtained from the patient prior surgery. Experimental work with the human muscle cells was approved by the Helsinki Committee of the Israeli Ministry of Health (Yaffe, 1968; Sarig et al., 2010, 2006).

The muscle was minced with scissors, enzymatically dissociated, at 37°C, with TrypLE (GIBCO 12604-013) for 30 min, and centrifuged at 2,500 rpm for 5 min (Yaffe, 1968; Sarig et al., 2010, 2006). The cells were collected, and trypsinization of the remaining undigested tissue was repeated three more times by adding fresh trypsin solution. After centrifugation, the cells were suspended in proliferation medium BIO-AMF-2 (Biological Industries Ltd., Kibbutz Beit Haemek, Israel), collected, and filtered through a 70 μm Cell Strainer (Corning^®^ 70 μm Cell Strainer, White, Sterile, Individually Packaged, 50/Case (Product #431751), Sigma CLS431751-50EA) to yield a single-cell suspension. The cells were plated on uncoated flasks for 2 h to deplete the fibroblasts (and macrophages). Unattached cells were then collected and transferred to gelatin coated flasks (Gelatin from bovine skin, Sigma G9391 Type B, powder, BioReagent, suitable for cell culture) to yield myogenic cells.

### Fluorescence-Activated Cell Sorting (FACS)

After the isolated myogenic cells were harvested from the tissue culture flasks, samples were incubated with anti-human CD56-Phycoerythrin (PE) antibody (Merck KGaA, Darmstadt, Germany). The labeled cells were thoroughly washed twice in flow-buffer (5% FCS, 0.1% sodium azide in PBS). Cells were suspended in 0.5 ml PBS and analyzed by a FACSCalibur^TM^ flow cytometer using an argon ion laser, adjusted to an excitation wavelength of 488 nm (FACS; Becton Dickinson Immunocytometry System, San Jose, CA, United States). An isotype control was performed with mouse IgG2b-PE (Miltenyi Biotec Inc., Auburn, CA, United States), and specific staining was measured from the cross point of the isotype with the specific antibody graph.

### Gene Cloning and Lentiviral Preparation

Human BDNF, GDNF, IGF-1, and VEGF genes were amplified from the pBluescript plasmids which were purchased from Harvard Institute of Proteomics, Boston, MA, United States, using Plasmid Midi Kit (Qiagen, Valencia, CA, United States) (Dadon-Nachum et al., 2012; Glat et al., 2016). Each of the four genes was inserted into the destination plasmid under the Cytomegalovirus (CMV) promoter in a recombinant reaction.

The constructs (3 μg) were co-transfected with the packaging plasmids (9 μg): pLP1, pLP2, and pLP/VSVG using Lipofectamine, 2000 (Invitrogen) into the 293 T producer cell line.

After incubation, the complexes were added to flasks containing 95% confluent hMPC cultured in an antibiotic free medium and incubated at 37°C in a 5% CO_2_ incubator. Each flask was transfected with a single NTF expression vector. Six hours later, the cell medium was changed to their standard growth medium.

The lentiviral titer was determined using the Lenti-X^TM^ p24 Rapid Titer Kit and the manufacturer’s recommended procedure (Cat. No. 632200, Takara Bio USA, Mountain View, CA, United States).

### ELISA Analysis

The hMPC were thawed and transduced with lentiviruses each containing BDNF, GDNF, IGF-1, or VEGF genes. Lentivirus containing green fluorescent protein (GFP) was used as a control. At the time of transduction, the cells were in passage 1 (P1). Transduction was done in multiplicity of infection (MOI) of 50. The presence of each of the secreted NTFs on the isolated and frozen cell supernatant was quantified 24 and 72 h after transduction using enzyme-linked immunosorbent assay (ELISA) kits (RayBiotech, Norcross, GA, United States). The assays were conducted according to the manufacturer’s protocols in duplicate, and absorbance was read at 450 nm using an ELISA reader (PowerWave X; BioTek Instruments, Winooski, VT, United States).

### Animals

Mice were maintained in 12-h-light/12-h-dark conditions in individually ventilated cages (IVC) with *ad libitum* access to food and water. All experimental protocols were authorized by the Tel Aviv University Committee of Animal Use for Research and Education. Every effort was made to reduce the number of mice used and minimize their suffering.

#### Sciatic Nerve Crush Mouse Model

The sciatic nerve crush model was performed on eight-week-old male C57BL/6J mice (*n* = 56; Harlan, Jerusalem, Israel). Just prior to surgery, mice were anesthetized with a mixture of ketamine-xylazine (100 mg/kg ketamine, 10 mg/kg xylazine). The left sciatic nerve was exposed, and a vessel clamp was applied for 30 s above the first branching of the nerve (Dadon-Nachum et al., 2012). A sham group of mice was included in which the sciatic nerve was exposed but not crushed.

#### Cell Transplantation

One day after SNI surgery, the genetically modified cells, at passage 3 (P3) resuspended in 100 μL saline, were injected into the lesion site. Two treatment groups were transplanted with a mixture of cells expressing all the NTF genes, i.e.: BDNF, GDNF, IGF-1, or VEGF, for a total amount of 10^6^ or 3 × 10^6^ cells (i.e., 2.5 × 10^5^ × 4 or 7.5 × 10^5^ × 4, respectively). The sham group was injected with 100 μL saline. The injured group comprised mice injected with saline, mice transplanted with 7.5 × 10^5^ hMPC harboring the GFP gene, and mice transplanted with 3 × 10^6^ non-modified hMPC (no significant difference was observed).

#### Behavioral Analysis

##### CatWalk test

The CatWalk XT 10.6 system (Noldus Inc., Netherlands) was used to assess gait recovery and motor function after SNI (Neumann et al., 2009; Vandeputte et al., 2010). This test involves monitoring each animal when it crosses a walkway with a glass floor illuminated along the long edge. Data acquisition was carried out using a high-speed camera, and paw prints were automatically classified by the software. The performance of each mouse was recorded three times, to obtain approximately 15 step cycles per mouse for analysis. Paw prints of each animal were obtained 3, 7, and 13 days after surgery.

##### Hot-plate test

Antinociception in the SNI model was assessed by the hot-plate test (Polt et al., 1994) 6 days post-SNI. Animals were placed on a hot surface, which was maintained at 55 ± 0.5°C. The time (in seconds) between placement and licking of the mice hind paws or jumping (whichever occurred first), was recorded as the response latency. A 20 s cut-off was used to prevent tissue damage.

#### *In vivo* Imaging

CRI Maestro^TM^ non-invasive fluorescence imaging system was used to follow the cells 2, 5, and 12 days following hMPC-GFP transplantation (the right sciatic nerve was crushed 1 day before cell transplantation, as described above). The area of interest was shaved and mice were anesthetized using ketamine-xylazine mixture and placed inside the imaging system. A band-pass filter appropriate for the fluorochrome of interest (GFP; Ex 445–490 nm, Em 515 longpass filter; acquisition settings 500–720) was used for emission and excitation light, respectively. Mice autofluorescence and undesired background signals were eliminated by spectral analysis and linear unmixing algorithm.

#### Gastrocnemius Preparation and Neurotrophic Factors Measurements

Five days after SNI (4 days after cell transplantation), 3 × 10^6^ hMPC-NTF treated mice (*n* = 3) and hMPC-GFP treated mice (*n* = 3) were sacrificed using CO_2_. Gastrocnemius muscles of both hind paws of each mouse were quickly removed in order to evaluate NTFs secretion from the tissues. Tissues were snap frozen in liquid nitrogen then transferred to -80°C until analysis.

##### Protein extraction

Tissues were thawed, and total protein was produced as previously described. Protein concentration was determined using the bicinchoninic acid (BCA) kit (Thermo Scientific, Rockford, IL, United States).

##### Quantification of NTFs levels using ELISA

The presence of each of the secreted NTFs was quantified using ELISA specific kits (RayBiotech, Norcross, GA, United States). The assays were conducted according to the manufacturer’s protocols in duplicate, and absorbance was read at 450 nm using an ELISA reader (PowerWave X; BioTek Instruments, Winooski, VT, United States).

### Statistical Analysis

The results are expressed as means ± standard error (SE). Statistical analysis was performed using unpaired Student’s *t*-test for the direct comparison between two groups. Statistical analysis of data sets was carried out with the aid of GraphPad Prism for Windows (GraphPad Software, La Jolla, CA, United States).

## Results

### Characterization of the Human Myogenic Cells

**FIGURE 1:**
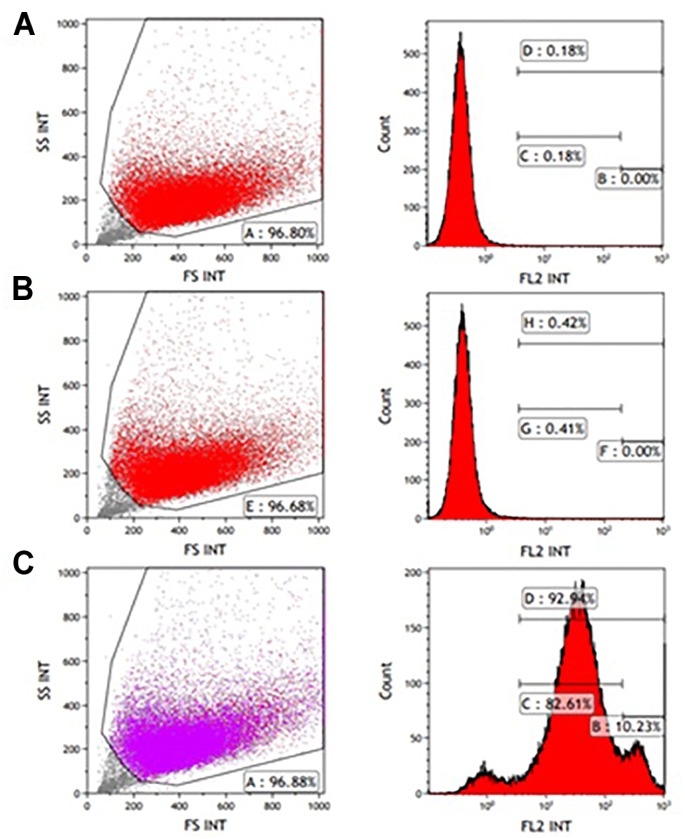
FACS analysis of CD56 expression in P1 myogenic cells. CD56-positive cells were resolved using light scattering (FS, forward scatter; SS, side scatter). **(A)** Incubation without antibodies was used as a baseline. **(B)** Staining for non-specific mouse immunoglobulin G (IgG) isotype fluorescence was used as a control. **(C)** 92.94% of the isolated cells express the CD56 surface marker related to human myogenic cells according to the peak area shown following staining with anti-human CD56-Phycoerythrin (PE) antibody.

**FIGURE 2:**
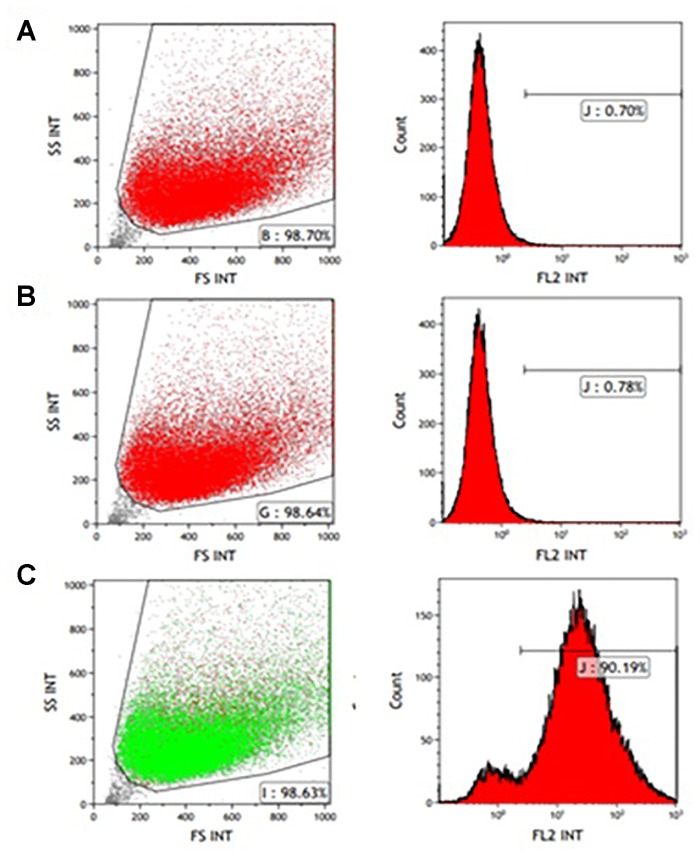
FACS analysis of CD56 expression in P3 myogenic cells. CD56-positive cells were resolved using light scattering (FS, forward scatter; SS, side scatter). **(A)** Incubation without antibodies was used as a baseline. **(B)** Staining for non-specific mouse immunoglobulin G (IgG) isotype fluorescence was used as a control. **(C)** 90.19% of the isolated cells express the CD56 surface marker related to human myogenic cells according to the peak area shown following staining with anti-human CD56-PE antibody.

CD56 antigen neural cell adhesion molecule (NCAM) is a known cell surface marker of human myogenic cells (Capkovic et al., 2008). Therefore, CD56 expression on the muscle-derived cells was examined by FACS analysis following labeling with mouse anti-human CD56- PE antibody. A high percentage of cells expressed the CD56 marker from passages P0-P5. Figure 1C, 2C show that 92.94 and 90.19% of the isolated cells in P1 and P3, respectively, expressed the marker. Incubation without antibodies was used as a baseline (Figure 1A, 2A), and staining for non-specific mouse immunoglobulin G (IgG) isotype fluorescence was used as a control (Figure 1B, 2B). Microscopic inspection revealed that the majority of the cells had a myogenic morphology, and only a few fibroblasts were observed, confirming that highly pure population of hMPC was established from the muscle biopsy.

### Characterization of the Transfected Human Muscle Progenitor Cells

**FIGURE 3:**
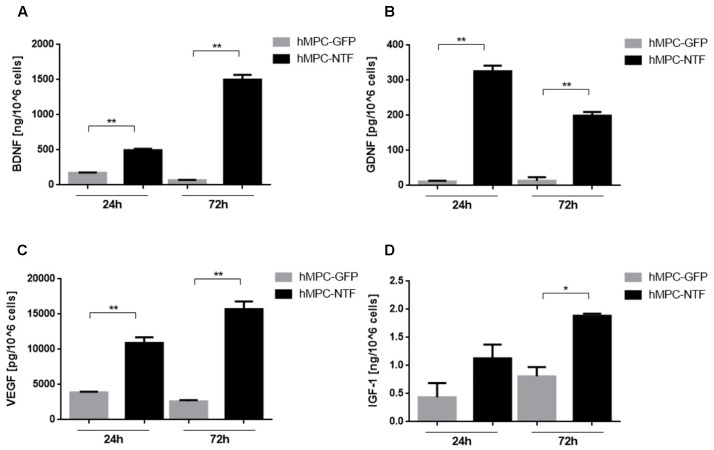
Genetically modified human myogenic cells secrete neurotrophic factors (NTFs). Levels of NTFs were measured using ELISA kits 24 and 72 h after the hMPCs were transduced with lentiviruses containing a gene for expressing either **(A)** BDNF, **(B)** GDNF, **(C)** VEGF, or **(D)** IGF-1. The levels of NTFs secreted from hMPCs harboring the GFP gene were measured as control values. The data are presented as mean ± SEM, ^∗^*P* < 0.05, ^∗∗^*P* < 0.01, one-tailed *t*-test.

The expression and secretion of NTFs from hMPC-transfected cells were assessed using ELISA analysis. The genetically manipulated hMPCs were found to express high levels of BDNF, GDNF, VEGF, or IGF-1. According to the ELISA kits, levels of NTFs were found to be 495.1 ± 21.3 ng and 1500 ± 68.8 ng of BDNF, 325.5 ± 16 pg and 199.2 ± 10.5 pg of GDNF, 10906 ± 802.9 pg and 15709 ± 1093 pg of VEGF, and 1.13 ± 0.24 ng and 1.88 ± 0.04 ng of IGF-1 per million cells, 24 and 72 h after cell transduction. In contrast, levels of NTFs secreted from GFP-transfected hMPC were significantly lower (Figure 3).

### Transplantation of Genetically Modified hMPCs Expressing NTFs Improved Motor Function and Gait Pattern in Sciatic Nerve Injury Mouse Model

To assess the effect of hMPC-NTF on recovery of nerve damage, mice were injected with cells 1 day after sciatic nerve crush surgery. The motor recovery effect was evaluated by parameters obtained from the CatWalk XT system on days 3, 7, and 13 after surgery.

**FIGURE 4:**
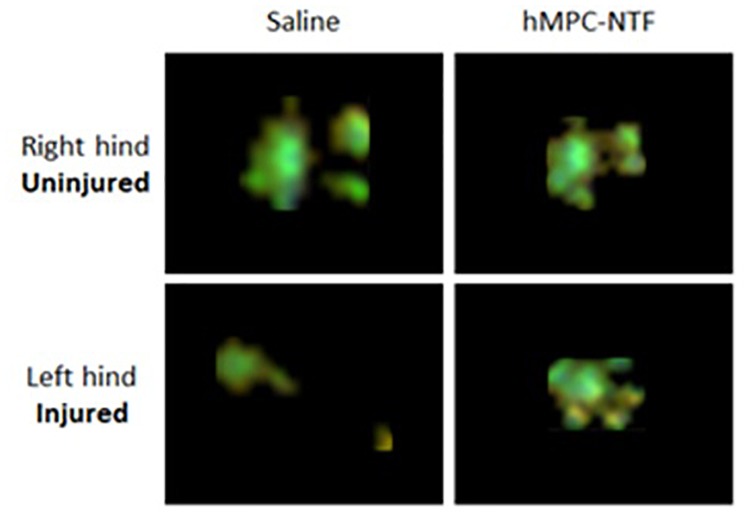
Illustration of gait pattern improvement following transplantation of genetically modified hMPCs expressing NTFs after SNI. Representative images of paw prints, acquired using CatWalk XT system, 7 days after SNI without treatment (right) or with 3 × 10^6^ hMPC-NTF treatment (left).

Figure 4 illustrates improvement in the gait pattern of the injured mice treated with 3 × 10^6^ hMPC-NTF by displaying two representative images of left hind paw after sciatic nerve crush, assessed 7 days after injury. The bottom right image in Figure 4 shows an exemplary footprint from the injured mice group treated with 3 × 10^6^ hMPC-NTF, and the left image is an exemplary footprint from the untreated injured group.

The maximum tread intensity, at maximum contact, and the paw print area parameter were quantified. Data from all groups were normalized to the average data of the naïve control group (*n* = 6).

**FIGURE 5:**
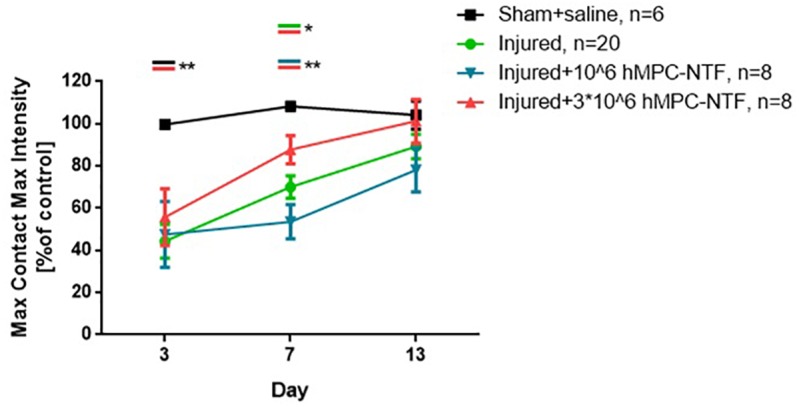
Transplantation of genetically modified hMPCs expressing NTFs improved motor function after SNI. Left hind paw maximum tread intensities, at maximum contact, were acquired using the CatWalk XT system on days 3, 7, and 13 post-SNI. These values were compared to those of naïve control mice, whose function was considered 100%. The data are presented as the relative mean function ± SEM of n mice per treatment group. ^∗^*P* < 0.05, ^∗∗^*P* < 0.01, one-tailed *t*-test.

**FIGURE 6:**
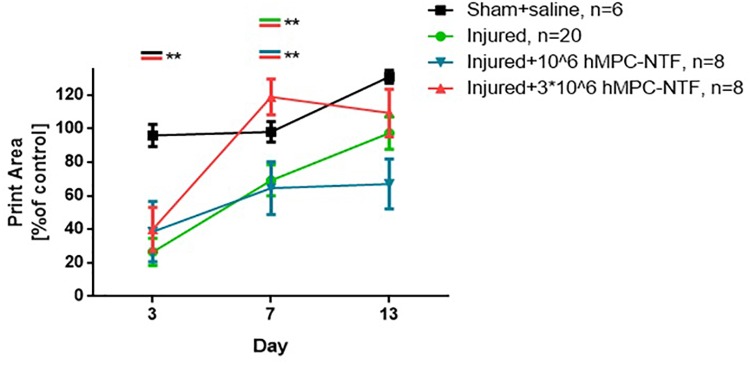
Transplantation of genetically modified hMPCs expressing NTFs improved gait pattern after SNI. Left hind paw print areas were acquired using CatWalk XT system 3, 7, and 13 days post-SNI. These values were compared to those of naïve control mice, whose function was considered 100%. The data are presented as the relative mean function ± SEM of n mice per treatment group. *^∗∗^P* < 0.01, one-tailed *t*-test.

The values obtained for both of these parameters were similar in control and sham groups. These values were found to be significantly lower in the injured groups as compared with the sham group, 3 days post-SNI, regardless the treatment given (Figure 5, 6).

However, 7 days post-surgery, the values were significantly different in the group of injured mice treated with 3 × 10^6^ hMPC-NTF, as compared to both of the other injured groups, either treated with 10^6^ hMPC-NTF, or untreated mice (Figure 5, 6). Recovery of motor function and gait pattern for the mice transplanted with 3 × 10^6^ hMPC-NTF was better at this time point, and their motor function and gait pattern resembled those of the sham group. Nevertheless, the motor function and gait pattern of untreated injured mice recovered to some extent due to spontaneous regeneration of the sciatic nerve. Notably, transplantation of 10^6^ hMPC-NTF didn’t have a significant effect on the injured mice.

Thirteen days after surgery the significant difference in motor function and gait pattern between the experimental groups disappeared due to spontaneous regeneration of the sciatic nerve.

### Transplantation of Genetically Modified hMPCs Expressing NTFs Improved Sensory Deficits in the Sciatic Nerve Injury Mouse Model

**FIGURE 7:**
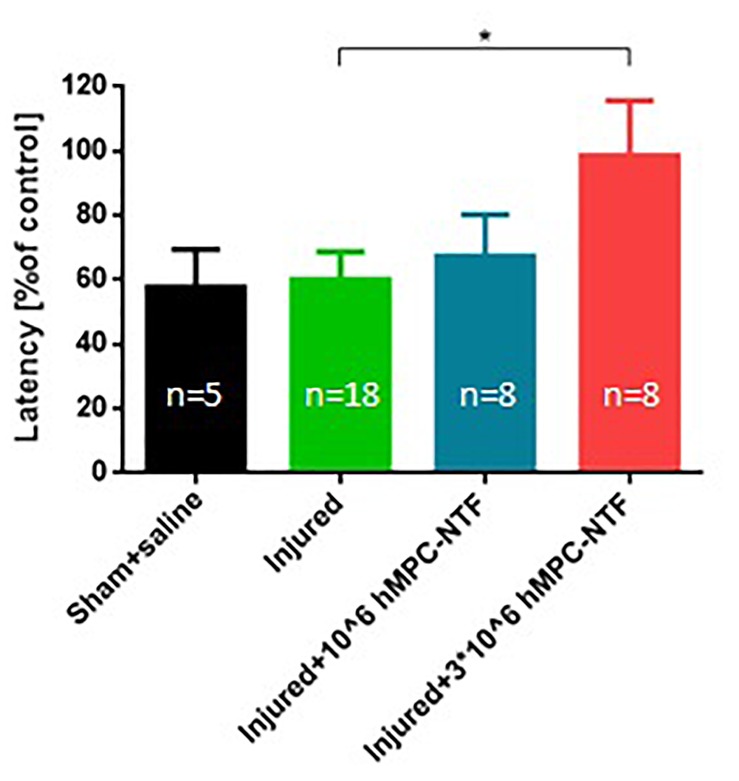
Transplantation of genetically modified hMPCs expressing NTFs improved sensory deficits after SNI. Nociceptive threshold of the left hind paw was tested by measuring latency of analgesic response in the hot-plate test. These values were compared to those of naïve control mice, whose function was considered 100%. The data are presented as the relative mean response ± SEM of n mice per treatment group, ^∗^*P* < 0.05, one-tailed *t*-test.

Sensory fiber regeneration was evaluated using the hot plate test on day 6 after SNI. Injured mice treated with 3 × 10^6^ hMPC-NTF were significantly less sensitive to hot plate exposure, than untreated injured mice, and their response resembled those of naïve control mice (Figure 7). These results indicate a palliative effect of the treatment on the injured paws.

### *In vivo* Imaging of Transplanted Cells Was Correlated With Behavioral Results

**FIGURE 8:**
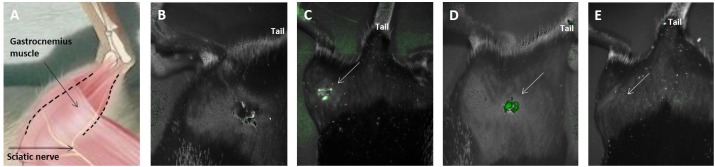
*In vivo* imaging of transplanted cells was correlated with behavioral results. hMPC-GFP cells were tracked after transplantation into the right gastrocnemius muscle using CRI Maestro^TM^ non-invasive fluorescence imaging system. **(A)** Schematic illustration of the posterior right limb of a mouse, in which the sciatic nerve and the gastrocnemius muscle are indicated to assist in understanding the following images. **(B)** A negative control mouse, to which cells have not been transplanted. **(C,D)** hMPCs marked with green fluorescence protein (GFP) were detected within the gastrocnemius muscle **(C)** 2 and **(D)** 5 days after transplantation. **(E)** Image taken 12 days after transplantation, in which the cells are no longer detectable within the tissue. White arrows in **(C–E)** mark the transplantation area.

Using CRI Maestro^TM^ non-invasive fluorescence imaging system hMPC-GFP were examined 2, 5, and 12 days after transplantation into the gastrocnemius muscle. As seen in Figure 8C,D cells were present in the tissue 2 and 5 days after transplantation. After 12 days, it was no longer possible to detect the cells (Figure 8E). A negative control mouse (to which cells were not transplanted) was used to verify that the detected fluorescence was not due to autofluorescence (Figure 8B). Figure 8A schematically illustrates the posterior right limb of a mouse in order to assist in understanding the animal’s position in the images mentioned above.

### Intramuscular Injection of Genetically Modified hMPCs Expressing NTFs Resulted in an Increased NTF Expression Levels

Using an ELISA analysis, the NTF expression levels in the gastrocnemius muscle of the 3 × 10^6^ hMPC-NTF treated mice (*n* = 3) and hMPC-GFP treated mice (*n* = 3), were assessed 4 days after cell transplantation. According to the ELISA kits, levels of NTFs were found to be 4.92 ± 1.13 ng of BDNF, 4.16 ± 0.1 pg of GDNF, 7.44 ± 0.69 pg of VEGF, and 21.51 ± 5.91 ng of IGF-1, per mg protein extracted from the left hind gastrocnemius, injected with hMPC-NTF. In contrast, NTF expression levels from the left hind gastrocnemius injected with hMPC-GFP were lower than in the mice injected with hMPC-NTF (significantly lower for GDNF, VEGF, and IGF-1). NTF expression levels from the untreated right hind gastrocnemius were mostly undetectable.

## Discussion

Peripheral nerve injury is a common condition, ranging from mild to severe injury. Within several weeks a natural healing process begins to take place. Nevertheless, approximately 100,000 patients undergo peripheral nerve surgery in the United States and Europe annually, due to severe damage or continuous pain (De Albornoz et al., 2011). Functional recovery is frequently poor after peripheral nerve injury, and, except for surgical cases, the sole therapeutic options are palliative care, including painkillers and anti-inflammatory drugs to relieve the pain (Gordon et al., 2011).

The object of this study was to evaluate whether ectopic transplantation of human MPC expressing the NTF genes BDNF, GDNF, VEGF, and IGF-1 can alleviate sensory and motoric deficits identified in a mouse model of SNI.

Trophic activities in muscle and nerve were shown to increase after lesions and blockade of nerve activity (Brown et al., 1991; Houenou et al., 1991; Bedi et al., 1992; Danielsen et al., 1994). In addition, muscle extract was shown to potently prevent motor neuron degeneration (Oppenheim, 1985; Oppenheim et al., 1988). These results suggest that the presence of trophic factors in the nerve and muscle is important for motor neuron survival and nerve regeneration (Naveilhan et al., 1997). Furthermore, Rabinovsky et al. (2003) suggested that actions of IGF-1 on peripheral nerve regeneration can be connected to both, its neurotrophic effects and to its myogenic effects.

The intracellular signaling mechanisms, stimulated by each one of the NTFs – BDNF, GDNF, VEGF, and IGF-1, involve binding to a specific receptor and initiation of the PI3/AKT signaling cascade, which promote cell survival (Wilker et al., 2005; Wang et al., 2007; Karar and Maity, 2011; Chen et al., 2013). Nevertheless, motoneuronal upregulation of NTFs including BDNF and GDNF was found to occur within 7 days of injury and to progressively decline thereafter (Boyd and Gordon, 2003). This decline was suggested as a likely factor that correlates with the reduction of regenerative capacity after severe nerve damage (Gordon et al., 2011). In addition, considering the properties of NTFs and their positive effect on regeneration and motor neuron support, the attempt to test for the provision of NTFs as a potential therapy for peripheral nerve injury is compelling.

It can be concluded that the results obtained in the present research, regarding the improvement of motor and sensory deficits of SNI using transplantation of hMPC-NTF to the gastrocnemius muscle of the injured limb, were the result of both the positive effects of NTFs, and of an ongoing delivery of NTF secretion by MPC.

These results reinforce those of two previous studies. The first, showed the use of myogenic cells ectopically expressing the NTFs – BDNF, GDNF, IGF-1, and VEGF, in treating SNI (Dadon-Nachum et al., 2012). This was the first study to show the synergistic protective effect of the four NTFs in supplying a nurturing environment to the injured nerve. However, since the study used rat cells, further work was needed to show that human cells can also provide the same therapeutic potential.

The second study demonstrated that direct injection of viral vectors expressing the four NTF genes can accelerate the regeneration of the sciatic nerve after injury (Glat et al., 2016). However, lentiviruses are a powerful tool which, in addition to being immunogenic, also have the potential for being oncogenic, infectious, and can cause other transformative changes to infected cells (Schlimgen et al., 2016). Therefore, they are not a currently applicable approach in the clinic.

The results presented in this paper further support and reinforce findings of previous studies, suggesting a synergistic effect of the four NTFs for sciatic nerve reconstruction after injury. The use of MPC as a delivery system of the NTFs close to the site of damage has a myogenic effect that may, in and of itself, contribute to nerve recovery. It should be emphasized that the use of human cells as a treatment for mice shows an immunocompetent property of the treatment. The use of hMPC-NTF demonstrates the feasibility of a treatment paradigm with safe biological characteristics to the human body, which can lead to rapid, high-quality healing of damaged peripheral nerves due to modifications, resulting in an overexpression of NTFs.

## Author Contributions

RG, FG, TB-Z, UD, DY, and DO designed the experiments. RG and TB-Z performed the experiments. RL and AP contributed the muscle biopsy. RG, FG, DY, and DO wrote and edited the manuscript.

## Conflict of Interest Statement

FG and UD are employee of SCM. DY and DO hold patents related to engineered MPC which is assigned to Ramot at Tel Aviv University and Yeda at the Weizmann Institute of Science, and licensed to SCM. This does not alter our adherence to “Frontiers in Neuroscience” policies on sharing data and materials. The remaining authors declare that the research was conducted in the absence of any commercial or financial relationships that could be construed as a potential conflict of interest.
